# Prevention and Treatment of Hardware-Related Infections in Deep Brain Stimulation Surgeries: A Retrospective and Historical Controlled Study

**DOI:** 10.3389/fnhum.2021.707816

**Published:** 2021-08-26

**Authors:** Jiping Li, Wenjie Zhang, Shanshan Mei, Liang Qiao, Yunpeng Wang, Xiaohua Zhang, Jianyu Li, Yongsheng Hu, Xiaofei Jia, Yuqing Zhang

**Affiliations:** ^1^Department of Functional Neurosurgery, Beijing Institute of Functional Neurosurgery, Xuanwu Hospital, Capital Medical University, Beijing, China; ^2^Department of Neurology, Xuanwu Hospital, Capital Medical University, Beijing, China

**Keywords:** deep brain stimulation, implantable pulse generator, infection, hardware-related complication, treatment of infection

## Abstract

**Background:**

Hardware-related infection in deep brain stimulation (DBS) is one of the most commonly reported complications frequently resulting in the removal of implantable pulse generator (IPG).

**Objective:**

The aim of this study was to establish a useful strategy to better prevent and treat those infections and to improve the preservation rates of IPG.

**Methods:**

We conducted a retrospective and historical controlled study of all adult patients (≥18 years old) who had undergone initial DBS implantation at a single center. All participants were enrolled in the control group (between June 2005 and June 2014) or intervention group (between July 2014 and May 2019) based on their surgery dates. We used the intraoperative irrigation with hydrogen dioxide solution in the intervention group. Based on the dates of diagnosis, patients with hardware-related infection after DBS were enrolled in group A (between June 2005 and June 2014) or group B (between July 2014 and May 2019). IPG-sparing algorithm (Isa) was applied for group B. The early-onset IPG infections of the control and intervention groups were evaluated. The IPG preservation rates in both groups A and B were statistically analyzed.

**Results:**

Six cases of early IPG infection and subsequent IPG removal occurred in the control group, while none occurred after intraoperative usage of the hydrogen dioxide in the intervention group. IPG preservation rate of infected cases in group B was significantly higher than that in group A (70% vs.16%, *p* = 0.004).

**Conclusion:**

The combined application of hydrogen dioxide solution and Isa seems to be an effective strategy to prevent IPG infection.

## Introduction

Deep brain stimulation (DBS) has been widely accepted as an effective treatment for a variety of movement disorders such as Parkinson’s disease (PD), essential tremor (ET), and dystonia. The applications of DBS continue to expand as it is also employed in a range of other refractory neurological or psychiatric conditions (Awan et al., 2009). DBS-related infection has been identified as one of the most serious complications, with the reported incidence ranging widely between 1.2 and 23% (Hamani and Lozano, 2006; Sillay et al., 2008; Miller et al., 2009; Bhatia et al., 2010; Hardaway et al., 2018). Management of hardware-related infections can be challenging and it often involves prolonged hospital stays, further surgery, and partial or even complete hardware removal. However, effective prevention and treatment strategies for hardware-related infection of DBS remain under-investigated.

The most common sites involved in infection after DBS included frontal, postauricular area, and infraclavicular region with related DBS hardware of intracranial lead, connection cable, and implantable pulse generator (IPG), respectively. The IPG-related infection was of particular concern to our patients given that the rechargeable IPG was widely used since the second half of 2014. The cost of this new IPG type accounted for about 80% of the cost of the whole DBS system and it was higher than that of earlier non-rechargeable models, which was not covered by our national healthcare insurance system. Some reported that complete removal of the whole DBS device was performed in most cases with infections around any part of the DBS system, despite initial attempts of localized treatment (Oh et al., 2002; Umemura et al., 2003; Constantoyannis et al., 2005; Bhatia et al., 2010; Chen et al., 2017). The IPG salvage attempts frequently failed due to recurrent infections, except for one study group suggesting reuse of IPG following sterilization with ethylene oxide (Gocmen et al., 2014). According to the National Health Industry Standard, hospitals have not been allowed to reuse IPG after sterilization on their own since 2014. Furthermore, usage of rechargeable IPG (15-year warranty) implies a sustained risk of IPG infection. Those practices highlight the need for regular assessment for any suspected infections, better before the IPG is contaminated.

To establish a better strategy to prevent and manage those hardware-related infections following initial DBS surgery and to improve the preservation rates of IPG, we conducted a retrospective and historical controlled study in our DBS population.

## Materials and Methods

### Study Design

**FIGURE 1:**
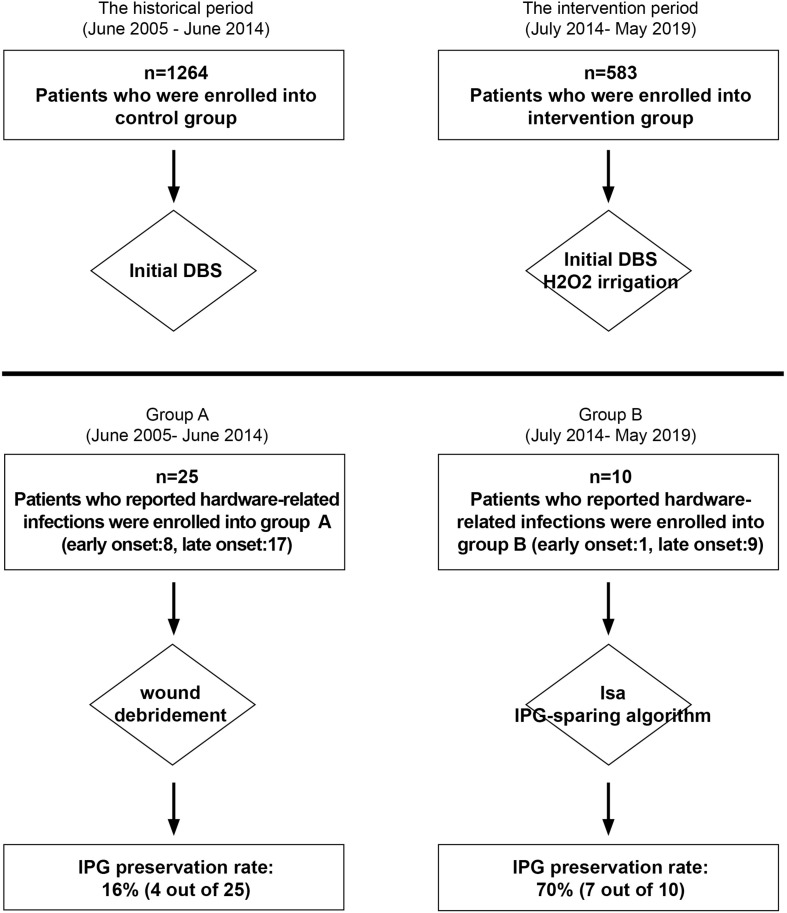
Enrollment of patients and flow diagram. DBS, deep brain stimulation; IPG, implantable pulse generator; H_2_O_2_, 3% solution of hydrogen peroxide, which was used at the IPG pocket to prevent the early and primary IPG infection.

We analyzed all adult patients (≥18 years old) who had undergone primary DBS implantation procedures performed in the period from June 2005 to May 2019 at a single center. As a historical controlled study, we reviewed their data retrospectively (Figure 1). Patients who underwent initial DBS implantation before June 2014 (control group) were compared with those who underwent DBS implantation after July 2014 (intervention group). Cases of hardware-related infection diagnosed before June 2014 (group A) were compared with those diagnosed after July 2014 (group B). We included only those patients who had received DBS implantation for the first time and excluded patients who had isolated IPG replacements. All infection rates were calculated based on the dates of diagnosis of infection. This study was approved by the Ethics Committee of Hospital and all patients signed consent documents.

### DBS Procedure

A multidisciplinary team selected the appropriate target nucleus: typically subthalamic nucleus (STN) or globus pallidus internus (GPi) for PD, ventralis intermedius (VIM) for ET, and GPi for dystonia and tics. Standard stereotactic techniques were used for all lead placements. MRI-CT fusion targeting was used for all patients, and intraoperative electrophysiology (microelectrode recording and macrostimulation) was used to help determine the final intracranial lead position. Lead implantations were performed under local anesthesia with monitored anesthesia care. For bilateral lead placements, the left lead was tunneled subgaleally to the right side to facilitate the placement of a single–dual channel IPG. Under general anesthesia, the IPG was placed in the infraclavicular region and connected to each intracranial lead by an extension cable in the same surgical setting. All procedures were performed by two neurosurgeons, namely, Zhang and Li.

### Definition and Classification of Infections

Only deep infections were considered in this study. Deep infections were defined as infections that extended into the subcutaneous layer and were in contact with at least one part of the DBS system (Piacentino et al., 2011).

Hardware infections of any part of the system were classified into early onset (<3 months) and late onset (>3 months). IPG infections were classified into primary and secondary infections based on their original sites. An infection presented initially in the subcutaneous pocket created for the IPG was classified as primary IPG infection. Secondary IPG infection was defined as an infection arising initially from frontal or postauricular and occurring at IPG pocket subsequently. The cellulites or purulent drainage could be seen and tracked in surgery, along the subcutaneous tunnel from the IPG pocket toward postauricular and frontal areas.

### General Antibiotic Regimen for Patients in the Control and Intervention Groups

All patients received prophylactic antibiotics intravenously 30 min before surgery, and postoperative antibiotic administration for 5 days following procedures. Ceftriaxone was administered as the prophylactic antibiotic for most patients (1 g intravenously 30 min before incision and 2 g intravenously per day postoperatively). Clindamycin was administered in cases of ceftriaxone allergy. Since July 2014, for all patients of DBS implantation, an additional 3% solution of hydrogen peroxide was used at the IPG pocket to prevent the early and primary IPG infection (Figure 1).

### Treatment of Hardware-Related Infections in Group A

We first attempted conservative treatments consisting of antibiotic treatment, wound debridement, and occasional scalp rotational flap. Decisions to remove hardware partially or completely were made for those patients following failure in conservative treatments. With or without hardware removal, patients were all managed with 2–4 weeks of intravenously administered antibiotics based on bacterial culture and sensitivity results.

### Treatment of Hardware-Related Infections in Group B

If infection presented originally from IPG pocket, antibiotic treatment, wound debridement, and IPG removal were performed. If infection occurred in the frontal or postauricular area, an IPG-sparing algorithm (Isa) was performed. Isa included the following three steps: (1) skin of the whole chest was prepped, and the IPG (with no signs of infection presented) was prophylactic ex-planted and reimplanted instantly into a new made pocket at contralateral infraclavicular region; (2) after prepping and draping again, hardware (intracranial leads and extensions) in the contaminated area was totally removed; and (3) 3 months later, if no clinical infection signs presented at the new IPG pocket, new DBS leads and extensions were implanted stereotactically and connected to the saved IPG through a new made tunnel at contralateral postauricular site. Patients were managed with the intravenous administration of antibiotics for 7 consecutive days following each of step (2) and step (3).

### Statistical Analysis

The gender, age at surgery, disease duration, disease categories, overall infection rate, and the number of early IPG infections of patients were compared between the control and intervention groups. IPG preservation rates, infection-related procedures, and accumulative hospital stay in group A and group B were statistically analyzed. The paired sample *t*-test, two-sample *t*-test, chi-square test, or Fisher’s exact test was used as appropriate for variables examined. *P* < 0.05 was considered statistically significant. Statistical analyses were performed by using SPSS 20.0 (SPSS Inc., Chicago, IL, United States).

## Results

### Patient Demographic of Control Group and Intervention Group

In total, 1,264 patients underwent DBS between June 2005 and June 2014 (control group), and 583 patients who underwent DBS between July 2014 and May 2019 (intervention group) were included in this study. Their demographics and diagnosis are reported in Table 1. There was no statistical difference in gender, age at surgery, disease duration, disease categories, or overall infection rate between the control and intervention groups. Six cases of early primary IPG infection and subsequent IPG removal occurred in the control group, while none occurred after intraoperative usage of the hydrogen dioxide in the intervention group. No intracranial abscesses were observed in both groups. The minimum follow-up of patients was 70 months (range: 70–178 months) in the control group and 12 months (range: 12–70 months) in the intervention group.

**TABLE 1:** Demographics of control group and intervention group.

	**Control group**	**Intervention group**
No. of DBS patients	1264	583
Male:female	740:524	358:225
Age at surgery (mean ± SD, years)	56.7 ± 13.4	58.8 ± 12.7
Disease duration (mean ± SD, years)	9.3 ± 7.6	8.4 ± 6.1
Diagnosis		
Parkinson’s disease	1119	534
Dystonia	72	23
Essential tremor	41	20
Tic	28	5
Others*	4	1
No. of rechargeable IPG patients (%)	49 (3.9%)	476 (81.6%)
Preventive measures against infection	antibiotic	antibiotic and H_2_O_2_ irrigation at IPG pocket
No. of overall infection (%)	25 (2.0%)	10 (1.7%)
No. of early primary IPG infection and IPG removal	6	0
Follow-up (range, months)	70–178	12–70

DBS, deep brain stimulation; IPG, implantable pulse generator; H_2_O_2_, 3% solution of hydrogen peroxide; others*, vegetative state, chorea, hemiballis, and cerebral palsy.

### Statistical Analysis of Infection in Both Group A and Group B

After the application of Isa (Table 2), the IPG preservation rate in group B was significantly higher than that in group A (70% vs. 16%, *p* = 0.004). The infection-related procedures in group B were significantly reduced than that in group A (2.2 vs. 3.6, *p* = 0.002). The accumulative hospital stay in group B was significantly shortened than that in group A (26.0 vs. 33.7 days, *p* = 0.008).

**TABLE 2:** Statistical analysis of infection in group A and group B.

	**Group A**	**Group B**	***P***
No. of hardware-related infection	25	10	
Early onset, <3 months (%)	8 (32%)	1 (10%)	0.235
Late onset, >3 months (%)	17 (68%)	9 (90%)	
Arising from IPG pocket (%)	9 (36%)	1 (10%)	0.218
Arising from frontal or postauricular area (%)	16 (64%)	9 (90%)	
IPG infection, *n* (%)	22 (88%)	3 (30%)	0.002
IPG removal, *n* (%)	21 (84%)	3 (30%)	0.004
IPG preservation, *n* (%)	4 (16%)	7 (70%)	0.004
Infection related surgery (per patient)	3.6	2.2	0.002
Infection related hospital stay (days per patient)	33.7	26	0.008

### Clinical Characteristics of Infection in Group A

**FIGURE 2:**
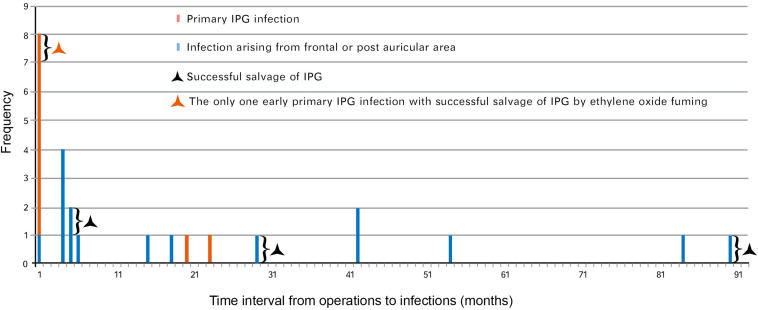
Hardware-related infections in group A (time interval to diagnosis of infection). Twenty-five cases of hardware-related infections were enrolled in group A (between June 2005 and June 2014) based on the date of diagnosis. Eight cases presented early-onset infection while 17 cases presented late-onset infection. IPG, implantable pulse generator.

Twenty-five hardware-related infections were diagnosed in group A and the characteristics of that group were described as early onset and late onset (Figure 2). Most (seven out of eight) of the early infective patients developed primary IPG infection. Of those seven patients, six presented recurrence of IPG infection after conservative treatment and finally resulted in IPG removal. The only one early primary infection with successful salvage of IPG was reported with sterilization of IPG using ethylene oxide (Figure 2).

The late infections appeared to be more common than early infections in this group (17 vs. 8). Of those 17 late infections, 15 were aroused from frontal and/or postauricular areas. Three frontal infections occurring at 5, 29, and 90 months postimplantation, respectively, were managed by intracranial lead removal and reimplanted 3 months later without infection recurrence (Figure 2). The other 12 infections spread and resulted in secondary IPG infection and IPG removal.

**FIGURE 3:**
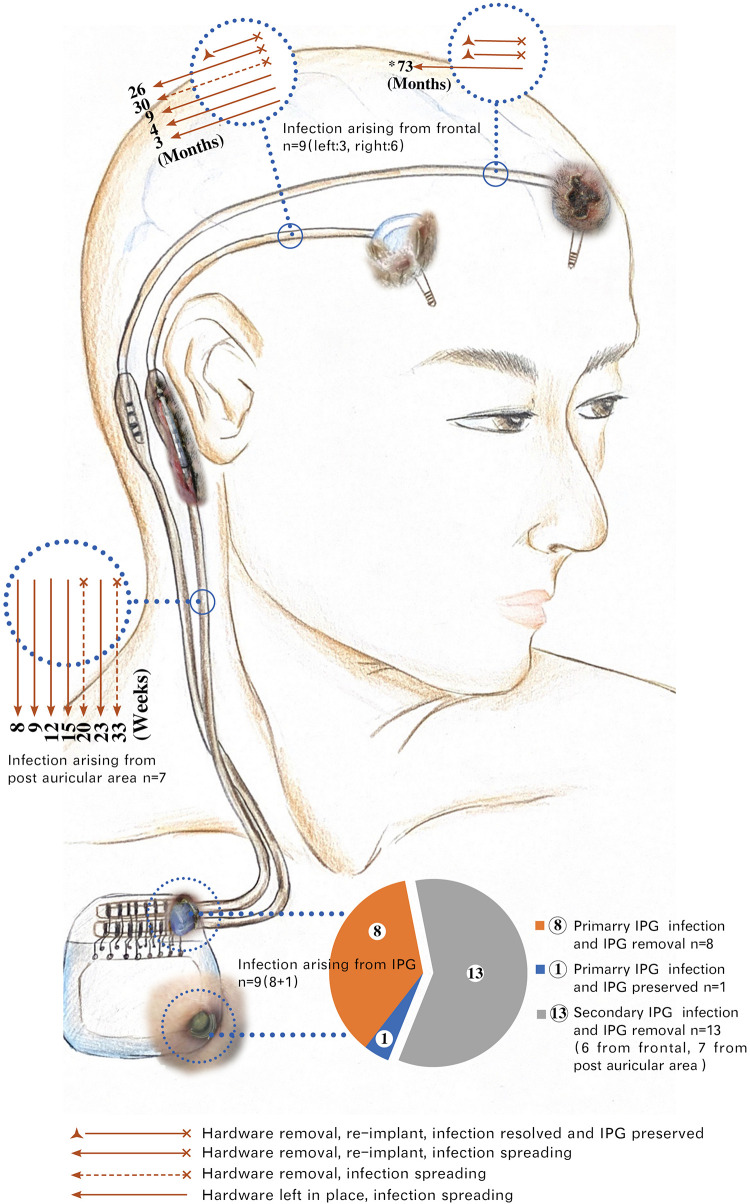
Spreading of hardware-related infections in group A. *73: The infection arising from the left head spread to the right postauricular area 73 months post-diagnosis of infection. This patient underwent five times of local incision debridement (including two times of rotational flap) before intracranial lead removal. Months: Time interval of infections spreading from frontal to postauricular area counted by months. Weeks: Time interval of infections spreading from postauricular area to infraclavicular region (IPG pocket) counted by weeks. Six infections of frontal origin and seven infections of postauricular origin finally spread to IPG. Among a total of 22 IPG infections, 21 were removed, with only 1 IPG remaining at a place after being sterilized with ethylene oxide. The exposed parts of the hardware in the picture were taken from real photos. IPG, implantable pulse generator.

In total, there were 22 IPG infections (9 primary and 13 secondary) resulting in 21 cases of IPG removal (Figure 3), and 4 out of 25 cases (16%) resulted in successful IPG preservation. An average of 3.6 infection-related procedures was performed and the accumulative hospital stay of patients was 33.7 days.

### The Acceleration in the Process of Infection Spreading in Group A

**FIGURE 4:**
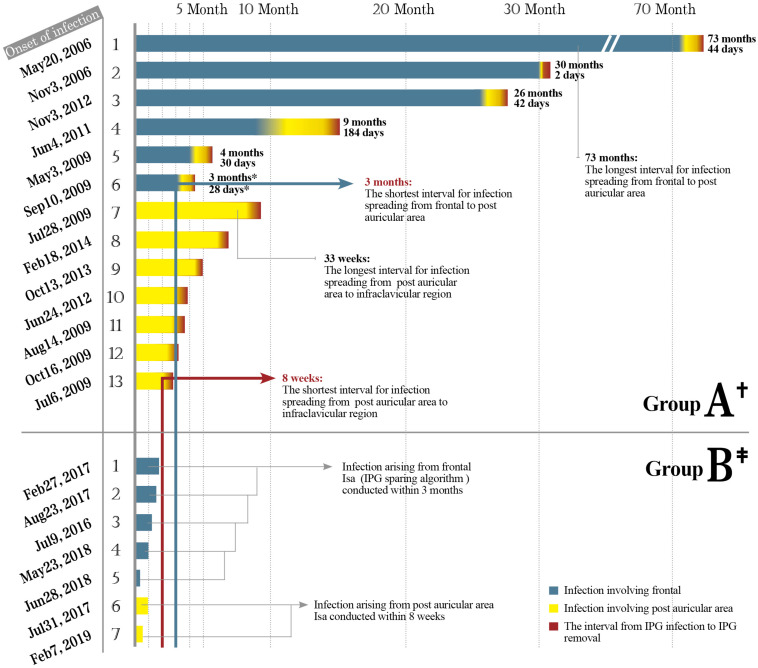
The safe time window for the application of Isa. Thirteen cases of infection spread to IPG in group A including six originating from frontal while seven originating from postauricular area. Seven cases of infection in group B underwent Isa within the safe time window. Months and days: The interval of infection spreading from frontal to postauricular area counted in months and subsequently to infraclavicular region (IPG pocket) counted in days. Significant difference in the interval duration was reported between two sections (Wilcoxon signed-rank test, *p* = 0.025). IPG, implantable pulse generator.

We found that the infection spreading presented an accelerating process for patients in group A (Figure 4). It took about 3–73 months for the pathogen to spread from head to postauricular. While it took as quickly as 2–184 days for the pathogen to spread from postauricular to infraclavicular region (IPG pocket). Significant difference in spreading duration was reported between two sections (Wilcoxon signed-rank test, *p* = 0.025).

### Clinical Characteristics of Infection in Group B

**FIGURE 5:**
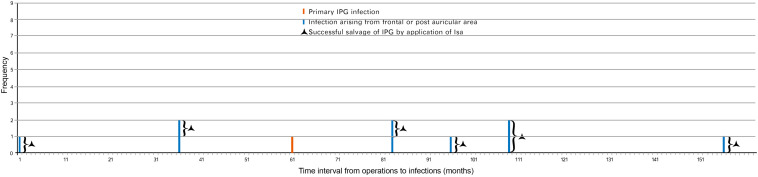
Hardware-related infections in group B (time interval to diagnosis of infection). Ten cases of hardware-related infections were enrolled in group B (between July 2014 and May 2019) based on the date of diagnosis. One case presented early-onset infection while nine cases presented late-onset infection. IPG, implantable pulse generator.

Ten hardware-related infections were diagnosed in group B (Figure 5). After Isa strategy was applied, seven infective cases were succeeded in preventing the infection from spreading down to IPG pocket and succeeded in IPG preservation. An average of 2.2 infection-related procedures was performed and the accumulative hospital stay of patients was 26 days.

### Safe Time Window for Application of Isa in Group B

As in group A, the interval for an infection spreading from frontal to postauricular area was 3–73 months. However, the interval for an infection spreading from postauricular to IPG pocket was 8–33 weeks (Figure 3). The safe time window was calculated based on the abovementioned minimal time interval. Therefore, for all seven patients with infection arouse from frontal and/or postauricular in group B, the IPGs were transimplanted to the contralateral chest subcutaneously within the safe time window (Figure 4), if only the infection was timely diagnosed.

### Adverse Effects

No operative complications related to reimplantation of intracranial lead occurred. No side effects of the application of intraoperative hydrogen dioxide solution, such as air embolism, were recorded.

## Discussion

The initial motivation for this study started in 2014 when there was a significant push to offer rechargeable IPG due to its advantage of a longer lifetime and less-frequent battery replacement (Waln and Jimenez-Shahed, 2014). However, the cost of this new IPG type is much higher than that of earlier non-rechargeable models and cannot be covered by our national healthcare insurance system. Many literatures have focused on the hardware salvage after infection (Sillay et al., 2008; Miller et al., 2009; Bhatia et al., 2010; Dlouhy et al., 2012; Atchley et al., 2019). However, the detailed description of effective preventive procedures for IPG infections in DBS surgery remains quite limited.

The increased surgical experience may reduce the incidence of infection; however, this viewpoint was not applied in our study. First, our surgical team had years of experience before data collection. In addition, in our long-term follow-up research, the more common infections were of late onset, which were attributable to patient-related factors rather than procedure-related factors. Therefore, our results showed that there was no significant difference in the overall hardware infection rate between the control and intervention groups.

### The Preventive Strategy for Primary IPG Infections

Primary IPG infections would probably arise early within 2 months, resulting in IPG removal despite multiple salvage attempts (Oh et al., 2002; Sillay et al., 2008; Miller et al., 2009). We observed the same in our study. Most early infections were associated with the procedure itself rather than the characteristics of patients. Sterilization of contaminated IPG with ethylene oxide fuming was suggested by a study group and succeeded in one of our patients of group A (Gocmen et al., 2014). However, this approach carried out by hospital rather than by manufacturer was not allowed according to the National Health Industry Standard (Regulation of Disinfection Technique in Healthcare Settings) since 2014. Moreover, there was no recommendation by the manufacturer regarding the approach to the problem. We did not employ junctive vancomycin powder intraoperatively because its beneficial was uncertain in literature (Waln and Jimenez-Shahed, 2014; Atchley et al., 2019; Bernstein et al., 2019).

Hydrogen peroxide has a broad spectrum of activity against gram-positive and gram-negative bacteria, bacterial spores, viruses, and yeast (Urban et al., 2019; Welman et al., 2019). There have been documented cases of hydrogen peroxide, resulting in air embolism from the formation of oxygen gas when used in closed cavities (Linley et al., 2012; Lu and Hansen, 2017). We would therefore advise that hydrogen peroxide should not be used with pressure or *via* intracranial. After junctive usage of hydrogen peroxide intraoperatively in the intervention group, no early primary IPG infection was observed with a minimum follow-up of 12 months. Although no statistically significant presented, the intraoperative usage of hydrogen peroxide did result in decreased cases of early primary IPG infection and subsequent IPG removal.

### The Preventive Strategy for Secondary IPG Infections

Secondary IPG infections resulted mostly from late infections, which were more common than early infections in our study. Analysis has concluded that most of the late infections are attributable to patient-related factors rather than procedure-related factors (Owens and Stoessel, 2008). Indeed, the risk of hardware-related infection can persist for up to several years after the patient was given an implant. There was a constant risk of IPG infection as long as the foreign materials remained *in situ* over the lifetime of patients (Barrett et al., 2018). Studies show that the infection risk was increasing as health conditions, such as hygiene habits, poor personal hygiene, and low cultural background, would have been worse particularly in advanced PD patients (Fischer et al., 2001; Bachmann and Trenkwalder, 2006; Owens and Stoessel, 2008; Sixel-Döring et al., 2010). This may also be the reason why the late infection was more likely to involve incision of frontal and postauricular areas. Compared with chest skin, the scalp hygiene was supposed to be more difficult to manage. And the skin around postauricular incision is more vulnerable to repeated friction. All those stress the necessity of a long-term follow-up and continued patient/caregiver education, particularly for patients implanted with a rechargeable and 15-year lifetime IPG.

Before Isa was applied, late infections would probably result in secondary IPG infection and IPG removal. Therapeutic strategy of initial infection was essential for preventing secondary IPG infection. There were usually several treatment options of initial infection as follows: (a) antibiotic treatment alone; (b) antibiotic therapy with wound incision and debridement; and (c) partial or complete removal of implanted DBS hardware. It was often surgeon dependent about which option should be initially conducted and the successful rates of hardware salvage differed widely among centers.

Based on our follow-up evaluation in group A, we found that most lead and/or connection infections would finally involve an IPG pocket (Figure 3). One possibility is that latent colonized bacteria of residual hardware may inoculate the newly implanted system, or translocate along the punctured tunnel, such that the infection showed in the least vascular IPG pocket. These results implied that the colonized bacteria in frontal or postauricular area would in great chance reach the IPG pocket and resulted in IPG removal if IPG-sparing management were not conducted promptly. While waiting for the therapeutic response of treatment in the traditional way, we would probably pass a valuable time window to protect the IPG in advance.

We considered altering our IPG salvage strategy by application of Isa in group B to (a) transimplant IPG before it was contaminated; (b) make a new tunnel to connect the new implanted lead and saved IPG to avoid the suspected organisms colonized in the old tunnel when reopening old incision. Isa strategy could significantly reduce secondary (late the majority) IPG infection if conducted within the safe time window (Figure 4). Those practices highlight the need for regular assessment for any suspected infections. Timely and effective communication among the neurosurgeon, patient, caregiver, and nurse practitioner is essential for the successful salvage of IPG.

### Limitations

As a retrospective and historical controlled study, this investigation did not involve all potential risk factors related to IPG infection. Thus, we did not have enough collection of variables to explore whether there were other factors contributing to IPG infection. The data might suggest to some degree that early IPG infection is frequently related to the surgical procedure itself while late IPG infection is commonly associated with the relevant factors of patients. Therefore, different potential prevention and treatment strategies are needed according to various characteristics of early and late infection.

Combined application of hydrogen peroxide solution and Isa could not reduce overall hardware infection since late infection accounted for the majority, which was assumed to be related to poor hygienic conditions and advanced age. Moreover, Isa may not be suitable for the elderly patients (>80) and those who cannot tolerate even a brief absence of stimulation delivered by the DBS device.

Early infections of our DBS patients were all documented, as their first follow-up for DBS programming was performed 3 months postoperatively in our hospital. However, there were chances of some cases of late-onset infection diagnosed and managed at other hospitals.

## Conclusion

The use of hydrogen peroxide can lower the incidence of primary IPG infection, and the Isa strategy can help prevent the occurrence of secondary IPG infection. Therefore, the combined use of the above two can effectively prevent and treat IPG infection, increase the preservation rate of IPG, and reduce infection-related procedures and hospital stays.

## Data Availability Statement

The original contributions presented in the study are included in the article/supplementary material, further inquiries can be directed to the corresponding author/s.

## Ethics Statement

The studies involving human participants were reviewed and approved by Ethics Committee of Xuan Wu Hospital. The patients/participants provided their written informed consent to participate in this study. Written informed consent was obtained from the individual(s) for the publication of any potentially identifiable images or data included in this article.

## Author Contributions

JPL, WZ, and YZ were the major contributors in writing the manuscript. SM, LQ, YW, XZ, JYL, and YH contributed to the diagnosis and treatment of the patients. JPL, WZ, SM, and XJ contributed to the data acquisition. JPL, WZ, and XJ contributed to the data analysis. LQ contributed to editing the manuscript. YZ reviewed the manuscript thoroughly and was accountable for all aspects of the work in ensuring that questions related to the accuracy or integrity of any part of the work. All authors read and approved the final manuscript.

## Conflict of Interest

The authors declare that the research was conducted in the absence of any commercial or financial relationships that could be construed as a potential conflict of interest.

## Publisher’s Note

All claims expressed in this article are solely those of the authors and do not necessarily represent those of their affiliated organizations, or those of the publisher, the editors and the reviewers. Any product that may be evaluated in this article, or claim that may be made by its manufacturer, is not guaranteed or endorsed by the publisher.
